# A new hybrid machine learning model for predicting the renewal life of patents

**DOI:** 10.1371/journal.pone.0306186

**Published:** 2024-06-26

**Authors:** Ashit Kumar, Pritam Ranjan, Arnab Koley, Shadab Danish

**Affiliations:** 1 OM&QT Area, Indian Institute of Management Indore, Indore, India; 2 Economics and Public policy Area, Indian Institute of Management Raipur, Raipur, India; Albert Einstein College of Medicine, UNITED STATES

## Abstract

In almost every country, patents need to be renewed multiple times after they are granted. A patentee assesses the value of the patent and then pays a renewal fee to keep it active for another stipulated period. The factors that characterize the value of a patent is subjective. This paper aims to address the research gap of building an accurate model for predicting the renewal life (often considered as a substitute for the patent value) of Indian patents, and identification of significant factors that influence the renewal life. This study uses an extensive data set collected from the Indian Patent Office for all granted patents filed between 1995 and 2005. The popular statistical and machine learning algorithms do not result in accurate predictive models, because the patent renewal life distribution (at least for the Indian patents) shows unusual spikes at the two extreme values, which makes the modeling task more challenging. We propose a new two-stage hybrid model by combining an efficient multi-class classifier and a binomial regression model for predicting the complex renewal data distribution. We conducted a comparative analysis of the proposed model with several state-of-the-art machine learning and statistical models. The results show that the proposed hybrid model gives 90% accuracy as compared to the best competitor which gives only 40% accuracy.

## 1 Introduction

Patents are economically and strategically important because the economic and technological value of patented innovations can influence future technological progress [1]. Because of their strategic and technological significance, firms, universities, and governments rely heavily on the ability to quickly identify high-value patents. Patented inventions serve as vital economic assets that contribute to the technological advancement of both companies and nations. According to Fasi [2], the assessment of patents and the recognition of patents with high value can furnish decision-makers with valuable information to guide their investment decisions in technology and patent applications. Furthermore, this aids policy makers in gaining insight into the trajectory of technology inside the nation, specifically in terms of its useful contributions. It also reflects on the efficacy of the patent system. In a given nation, when a substantial number of patent applications are deemed frivolous, it results in an increased deadweight loss and exposes the inefficiency of the patent system in effectively filtering out low-quality patents.

A patent is an exclusive legal authorization granted to the patent owner(s) for novel and non-obvious inventions for a limited period of time by patent offices which prevents others from using the invention without the innovator’s permission. A patent is instrumental in promoting innovation for the intellectual property ecosystem [3, 4]. The Indian Patent Act 1970 provisions the maximum life of a patent as 20 years from the filing date of the application, and the granted patent can be kept in force (remain active) till the maturity of the patent, that is, twenty years, by paying annual renewal fees. A non-payment of renewal fees within the due date or grace period results in the expiry or lapse of the patent rights. The *patent renewal life* is defined as the number of years a patent has remained active until it expires or matures. The patent renewal life depends upon the quality of the invention, the technology category, and the invented product’s market value [5, 6]. Svensson [7] study suggested that patents with a high-quality are more likely to be renewed and have a longer patent life. Indian patents have been the subject of extensive research lately.

In the last few decades, many researchers have used the renewal data to estimate the value and quality of patents. For instance, Pakes and Schankerman [8] developed a theoretical patent renewal model to estimate the appropriate revenue decay rate. A seminal work by Pakes [9] utilized patent renewal information to build a stochastic model and estimated the benefits of holding a patent in terms of revenue return over the life span of the patent. Sullivan [10] used the patent renewal framework to estimate the patent value distribution on patent rights in Britain and Ireland for the period 1852–76 and compared it with Pakes and Schankerman work. Bessen [11] inferred that a patent with longer renewal life had a higher patent value. Svensson [7] studied the effect of commercialization, quality of patents on patent renewal decisions, and his study shows that patents with a high quality level are more likely to be renewed. Danish et al. [12] used renewal data to estimate the private value of Indian patents and also compared the patent monetary value among various technology categories for Indian patents. Danish et al. [13] built a survival model using both parametric and semi-parametric approaches on Indian patent renewal data and suggested that technological scope and inventor size affect Indian patent life substantially.

An accurate prediction of patent renewal life is crucial as patent life is not only an indicator of patent value, quality, etc., but can also be utilized for the estimation of various technology transfer rates, and identification of determinants of patent life. To the best of our knowledge not much work has been done on the prediction modeling of patent renewal life, and this paper aims to address this research gap. The main objectives of this paper are to build a model that can accurately predict the renewal life of Indian patents, and identify significant factors that influence the renewal life.

**Fig 1:**
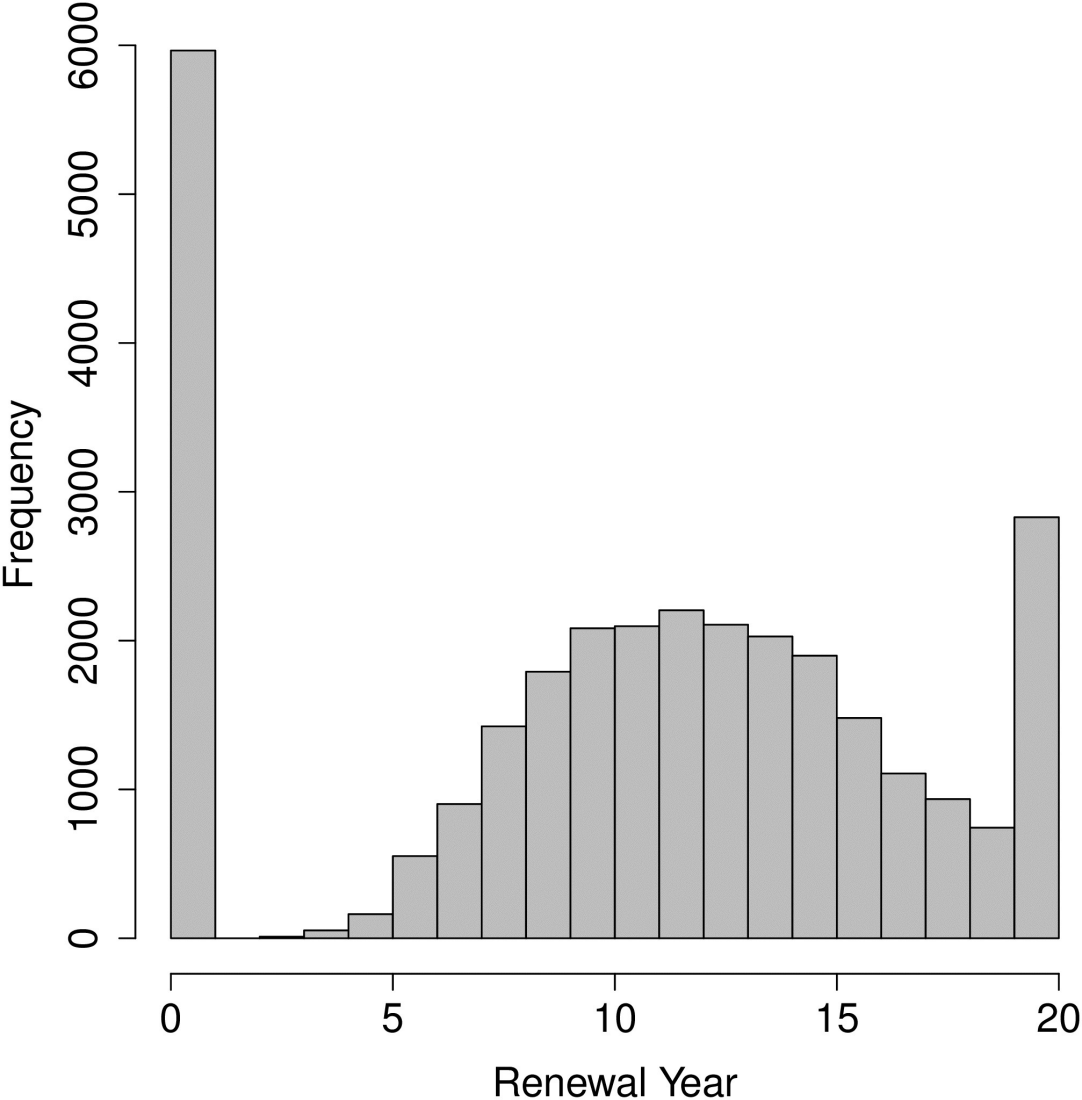
Histogram of the number of renewal years (for all 30372 patents).

This study uses patent level data collected from the Indian Patent Office (https://ipindiaservices.gov.in/publicsearch) and PatSeer (Gridlogics Technologies Pvt Ltd data) for all granted patents filed between 1995 and 2005. A quick look at the set of possible values of renewal life may indicate that a binomial distribution with total number of trials equal to 20 is an appropriate choice of its distribution, however, Fig 1 shows that the histogram of ‘Patent renewal life’ (Renewalyears) contains spikes at zero and twenty. Consequently, a binomial regression-based predictive model is not expected to give high accuracy. Of course, the two spikes can be explained conceptually, the spike at zero corresponds to a significantly high volume of never renewed patents, and the spike at twenty is due to the fact that an unusual push may be given to patents that are close to the maturity age.

The main contribution of this paper is to develop a predictive model that accounts for this unusual distribution of the patent renewal life. We propose a new two-stage hybrid model. The first stage builds an efficient classifier which predicts the label of a patent as “never renewed”, “expired” and “matured”. Subsequently, a generalized linear regression model is built for only predicting the renewal life of “expired” patents. For the first task, we have used a support vector classifier and for the latter, we used binomial regression model. For benchmarking, several state-of-the-art machine learning (ML) models have been used for building the predictive model. Additionally, we use the binomial regression model part to identify significant factors that affect the renewal life of the patents. Goodness of fit measure comparisons show that the proposed model outperforms by a significant margin.

The remaining paper is structured into five sections: Section 2 summarizes the data collected from the Indian Patent Office, and outlines the cleaning process which prepares the data for modeling. Section 3 briefly outlines the popular ML models used for benchmarking and the proposed two-stage hybrid model. Section 4 discusses the results by comparison of the goodness of fit measures for different models. Finally, Section 5 summarizes the outcome of this research and presents a few concluding remarks.

## 2 Exploratory data analysis

This section summarizes the data used for building the predictive models. We start with a variety of graphs and plots for gaining valuable insights, and then a few cleaning steps and transformation to prepare the data for our modeling purpose.

Patent renewal life is often used as an indicator of patent value and the quality of the invention. The patent level information data used for building the predictive model was collected from the Indian Patent Office website and PatSeer for all granted patents that were filed between 1 January 1995 and 31 December 2005. Most of the patent characteristics used in our predictive modeling have been discussed by many researchers in literature.

Table 1 presents the basic description, data type, and related references. Our data consists of 30372 patents with Renewalyear (renewal life) as the response variable and nine covariates: Filingyear, NumOfClaims, InventorSize, Familysize, TechScope, and GrantLag are continuous, whereas ownership is a binary, and Patentee Types and Techclass are categorical predictors. Patentee Types has three options—Individual, Institution and Firm, and Techclass refers to the patent technology groups identified by Danish et al. [12] in accordance with the four-digit International Patent Classification (IPC) 2008 Code, i.e., chemistry, electrical, instruments, mechanical and otherfields. Table 1 does not include Filingyear, because, we could not trace the usage of Filingyear in the literature as a covariate in predicting the value of a patent, however, as discussed in Section 4, our data shows a significant effect of Filingyear on the renewal life of a patent.

**Table 1:** Description of patent characteristics considered in building the predictive model.

Variable	Description (Independent Variables)	Variable type	Literature
Ownership	Resident (Indian) and non-resident	Binary	[12, 14]
Num-Of-Claims	No. of innovations claimed when filing the patent	Continuous	[6, 11, 15–17]
InventorSize	Inventor group size involved in developing the patent	Continuous	[12, 14, 18, 19]
FamilySize	No. of countries the patent application has been filed	Continuous	[14, 16, 20, 21]
TechScope	No. of technologies the patent belongs to	Continuous	[12, 14, 20, 22]
GrantLag	The time gap between the filing date and grant date	Continuous	[23, 24]
Patentee types	Type of patentee: Individual, Institution, Firm	Categorical	[12, 14]
Techclass	Technology group: chemistry, electrical, instruments, mechanical, and ‘otherfield’.	Categorical	[12, 14]

Table 2 presents the descriptive statistics for continuous variables in the data set. Note that NumOfClaims, FamilySize and TechScope have a very large range, and the sample means are relatively close to the minimum data value, which indicates that perhaps a few values are extremely large. Interestingly, the maximum value of GrantLag is 20, which is a bit weird, as the maximum patent life is 20 years in India. The maximum value of the FamilySize appears to be 381, which again looks suspicious as the total number of countries is less than 200.

**Table 2:** Descriptive statistics of the numeric variables (using all 30372 patents).

	Renewalyear	NumOfClaims	InventorSize	FamilySize	TechScope	GrantLag
mean	10.62	13.7	2.66	14.35	7.70	7.13
std	6.37	14.2	1.99	16.4	10.67	2.77
min	0	0	1	0	1	2
25%	7	6	1	2	1	5
50%	12	10	2	11	5	7
75%	15	17	4	19	9	9
max	20	422	24	381	231	20

**Fig 2:**
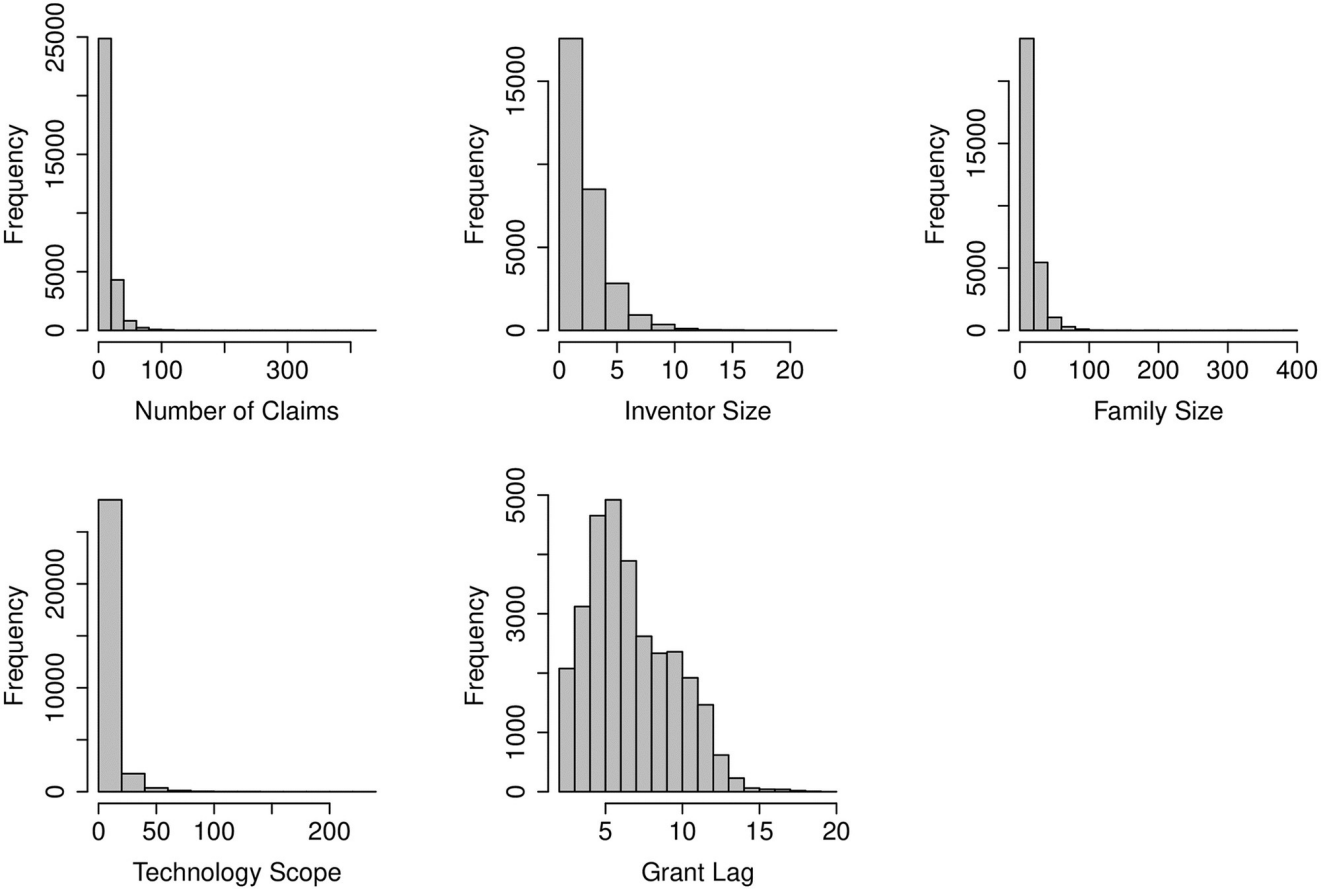
Frequency histograms of the numeric patent characteristics (using all 30372 patents).

The advent of digital technology has made it possible to simplify complex information by representing data in a visual form and creating more sophisticated and interactive visualisations of data / results, which assist in appropriate decision making. See Cao et al. [25] for a discussion on the current visualisation features of the Python Matplotlib library. There are many different types of data visualisation techniques, such as barcharts, linecharts, columncharts, piecharts, scatterplots, etc., each with its own strengths and weaknesses. We used histogram and boxplots for the basic data visualisation. Fig 2 presents the histograms of the continuous predictors. These are standard frequency histograms with equal width classes. The histograms show very right skewed distributions of NumOfClaims, InventorSize, FamilySize and TechScope with possible outliers.

**Fig 3:**
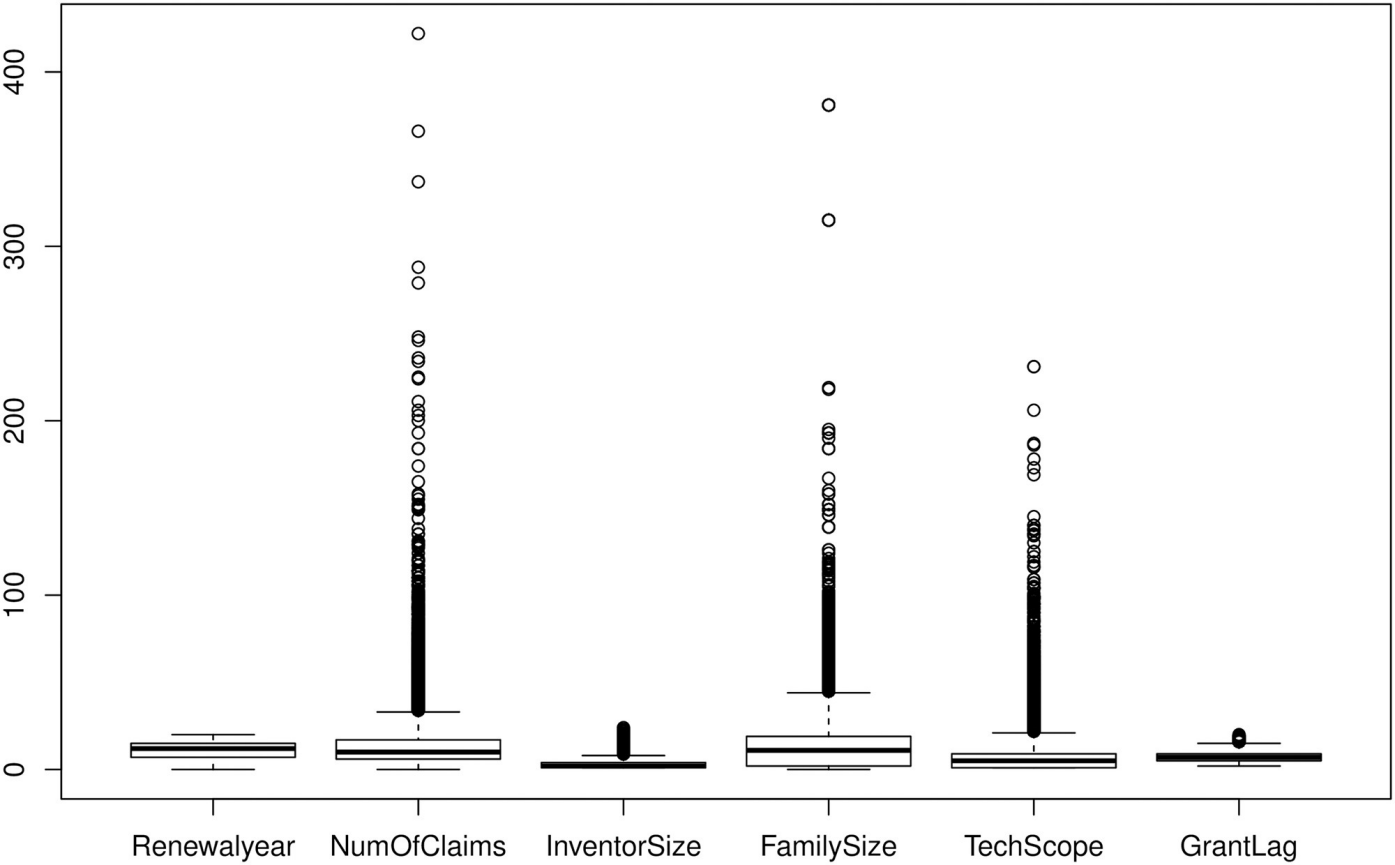
Boxplot of numeric patent characteristics (using all 30372 patents).

Nuzzo [26] suggested that visualisation techniques such as boxplot may provide better insights for outliers as compared to histogram and barcharts. The boxplots in Fig 3 clearly show that NumOfClaims, FamilySize and TechScope have heavy right tails, and the histogram in Fig 2 indicates that there are few patents for which granting process took more than 15 years from the filing date.

Data preparation for modeling is performed with the purpose of cleaning and converting the data into the most appropriate form, as the quality of the data used for modeling directly impacts the predictive performance of the model. We followed a four stage data cleaning process: (i) removal of extreme implausible data; (ii) transformation of data; (iii) outlier treatment and normalisation of data; and (iv) splitting into train and test data.

As per Table 2 and Figs 2 and 3 the distribution of NumofClaims, InventorSize, FamilySize and TechScope are very long right tailed, and the large values of GrantLag, close to 20, is also concerning. After due deliberation, we decided to drop a few patents from our study. In particular, (a) patents that took more than 15 years in the review process (i.e., GrantLag greater than 15) do not align with the general population and hence considered as outlier (b) a patent should have at least one innovation claimed, thus we dropped the patents with NumOfClaims equals zero; (c) the number of countries where the patent is filed should be at least one and unimaginable to be more than 200, and therefore the patents with FamilySize equals zero and more than 200 have also been removed from the predictive modeling exercise. As a result the data size reduced from 30372 to 30114.

**Fig 4:**
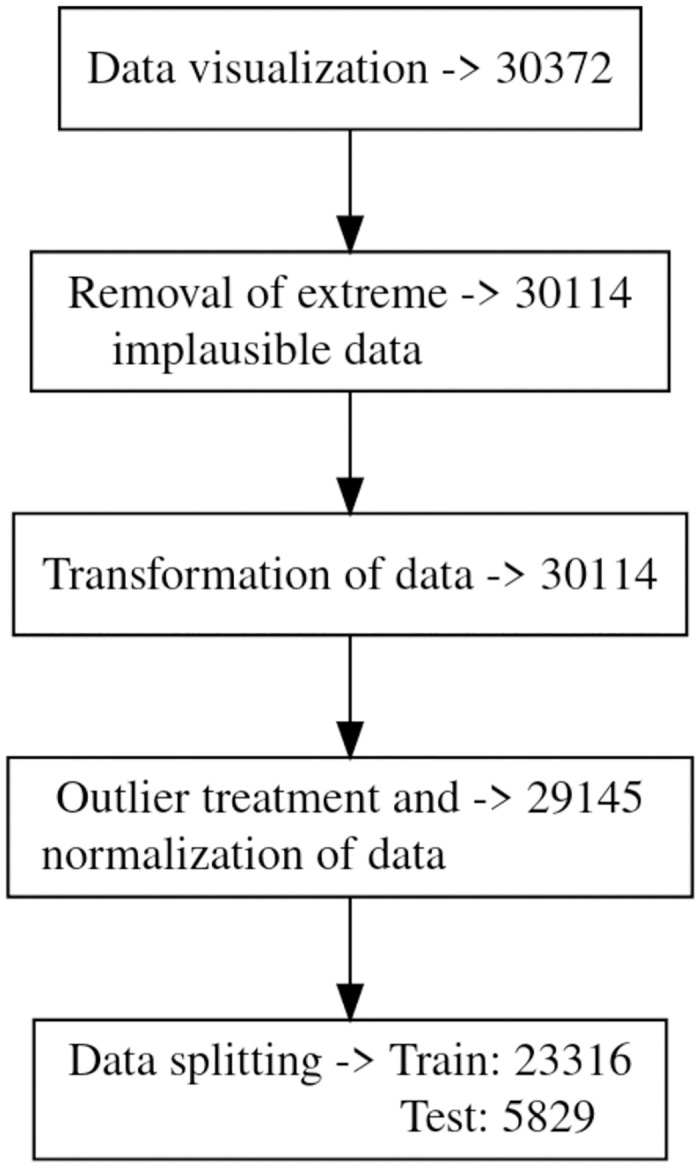
Flowchart of the change in data count due to different steps of cleaning of the data.

As predictors with symmetric or less skewed distributions are more suitable for a predictive models, we performed log-transformation on the continuous independent variables (NumofClaims, InventorSize, FamilySize, TechScope). The right-tailed extreme values of NumofClaims, FamilySize and TechScope were eliminated by dropping the top 1-percentile of the observations. At the end we had 29145 patents for building the predictive models. Fig 4 summarizes the steps of data cleaning and preparation along with the change in the counts.

**Fig 5:**
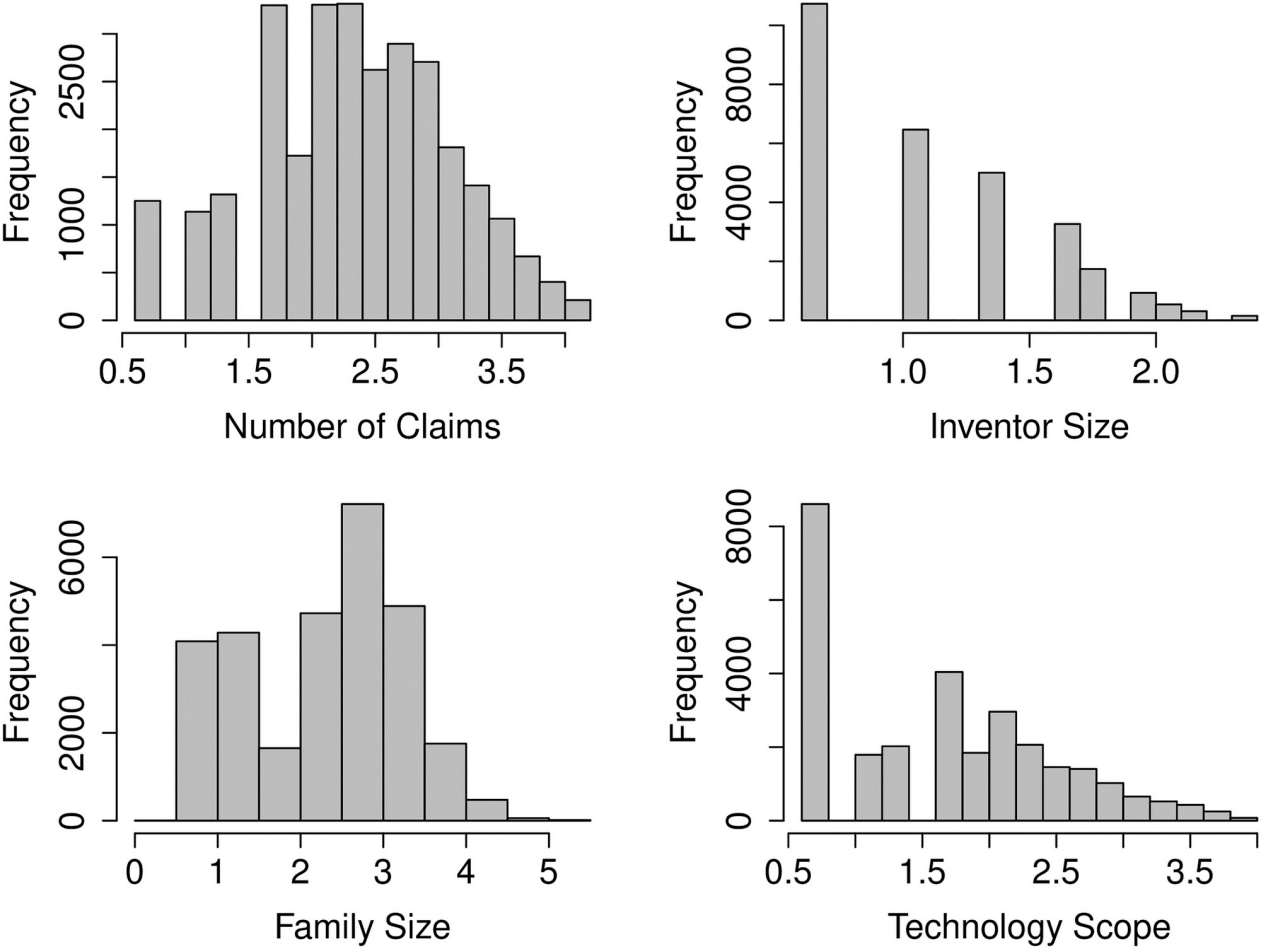
Histogram of numeric patent characteristics on log-scale (after the removal of unrealistic and outlier values, i.e., using 23145 patents).

**Fig 6:**
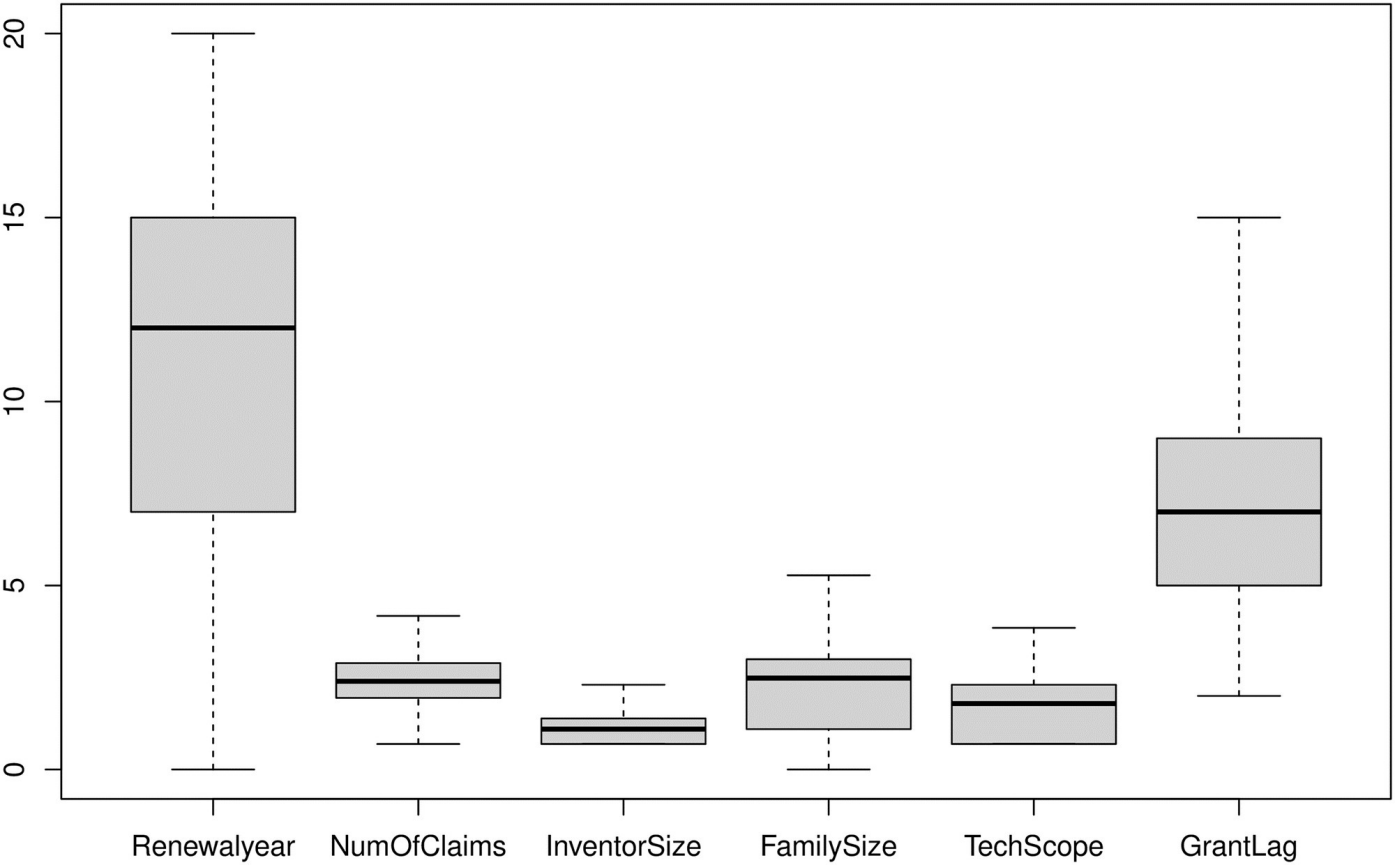
Boxplot of numeric patent characteristics on log-scale (after the removal of unrealistic and outlier values, i.e., using 23145 patents).

Figs 5 and 6 show the histograms and comparative boxplot of the log-transformed and cleaned data. Clearly the resulting data is outlier free and much more well-behaved for the modeling.

For computational stability in the modeling process all numeric predictors have been further scaled between [0, 1] using the min-max normalization technique. That is,

$$\begin{array}{c} X_{normalized}=(X-X_{min})/(X_{max}-X_{min}). \end{array}$$

Finally, splitting the full data into train and test is a critical component of building an accurate and reliable predictive model. The idea is to first fit the model on the train set only, and then use this fit to compare the predictive accuracy of the model with respect to both train and test data sets. The prime objective of this step is to prevent model overfitting with respect to the training data. The ratio of numbers of data points in train and test has been discussed extensively both in the Statistics and ML literature. For example, Joseph [15] studied the optimal ratio for data splitting and suggested $\sqrt{p}:1$ ratio for train and test in the linear regression modeling context, where *p* is the number of parameters. In general, practitioners follow Pareto principle and use simple random sampling without replacement to take 70-80% of the full data as training, and the complement set to be the test. In this study, we use 80% of the original data as train (*N*_*train*_ = 23316) and the remaining 20% as test (*N*_*test*_ = 5829).

## 3 The proposed methodology

In this section, we present several predictive models for accurate prediction of the renewal life of Indian patents using the patent characteristics discussed in Table 1. First we discuss an intuitive statistical regression model which will also serve as a benchmark for performance comparison. Then, several state-of-the-art ML models are presented. Finally, we propose a new two-stage hybrid model. The results of all these model fits for our patent data are discussed in Section 4.

### 3.1 Statistical regression model

The choice of a regression model is primarily driven by the data type of the response, typically denoted by *Y*. Since the renewal life of a patent (Renewalyear) is discrete and lies in a finite range, i.e., 0 ≤ *Y*_*i*_ ≤ 20, a binomial distribution is the most intuitive choice for characterizing the distribution of the renewal life.

Here, the corresponding binomial regression model would assume that *Y*_*i*_ ∼ *Binomial*(*m*, *p*_*i*_), with *m* = 20,

$$p_{i}=P\left(\text{renewal}\;\text{of}\;\text{the}\;\text{contract}\;\text{for}\;\text{the}\;\text{i-th}\;\text{patent}\right)$$

and *i* = 1, 2, …, *n*, where *n* is the number of patents in the train data. Furthermore, *p*_*i*_ is modelled with respect to the covariates (i.e., patent characteristics) as

$$\begin{array}{c} \text{log}(\frac{p_{i}}{1-p_{i}})={X_{i}}^{T}\beta =\beta_{0}+\sum_{j=1}^{p}X_{ij}\beta_{j}, \end{array}$$ (1)

where *X*_*ij*_ is the value of the *j*-th covariate for the *i*-th patent, *β*_*j*_ represents the respective regression coefficients, and *p* is the number of covariates. The model fitting was implemented via **Algopy** library in *Python*. In particular, we used *Newton conjugate gradient* (NCG) algorithm for the optimization purpose. NCG has speedy convergence capabilities of the Newton’s algorithm and the computational efficiency of the gradient decent [27, 28]. Model fitting yields $\hat{\beta }$—the estimate of the regression coefficients *β* = (*β*_0_, *β*_1_, …, *β*_*p*_), which in-turn gives $\hat{p}\left(X_{0}\right)=\text{exp}\left({X_{0}}^{T}\hat{\beta }\right)/\left(1+\text{exp}\left({X_{0}}^{T}\hat{\beta }\right)\right)$ for a given patent with characteristic vector *X*_0_, and the prediction equation for the renewal life of a patent would be $\hat{y}\left(X_{0}\right)=20\hat{p}\left(X_{0}\right)$. The standard error associated with the estimated parameters can be approximated as the square root of the diagonal of the inverse of the Hessian matrix,

$$SE\left({\hat{\beta }}_{j}\right)=\sqrt{h^{\left(jj\right)}},$$

where *h*^(*jj*)^ is the *j*-th diagonal element of *H*^−1^. In practice, the final value of *H*^(*k*)^ can be used for the inverse calculation.

### 3.2 Machine learning (ML) models

ML models have gained immense popularity for obtaining accurate predictions. Such models are extremely flexible and powerful in capturing complex relationship between the response and predictors. In this section, we present four state-of-the-art ML predictive models: random forest (RF), eXtreme Gradient Boosting (XGBoost), artificial neural network (ANN), and support vector regression (SVR).

#### 3.2.1 Random forest (RF)

Brieman et al. [29] pioneered the idea of tree-based models, which was later formalized as “Random Forest” (RF) in [30]. An RF is a collection of hundreds of independent random decision trees, which are built on the bootstrap samples of the data, and each node is split by finding the best variable-location combination using *m* < *p* randomly chosen predictors, where *p* is the total number of predictors. The predicted response is obtained by averaging the prediction from the member trees of the ensemble. See [31] for detailed methodology.

RF was implemented via **sklearn** library in *Python*. The hyper-parameters like the number of trees, depth of the tree, etc. were tuned using a grid-search based simulation study via train-test split. The best RF model (which resulted in the minimum root mean squared error) was obtained for 250 trees with the depth of each tree being equal to 3.

#### 3.2.2 Extreme gradient boosting (XGBoost)

XGBoost is a ensemble tree model based on the concepts of Newton Raphson and gradient boosting algorithms in which decision trees are updated sequentially by minimizing the residual error along with regularization methods for controlling the overfitting [32].

*Python* library **scikit-learn** was used to implement XGBoost model, and root mean squared error (RMSE) was used as the goodness of fit criterion for tuning the hyper-parameters: number of trees, depth of the tree, learning rate, and number of features used in each tree. In our case, a grid search with cross validation yielded the optimal model with 30 trees, max depth equal to 4, and learning rate equal to 0.2.

#### 3.2.3 Artificial neural network (ANN)

In current times, neural network-based models, popularly referred to as artificial intelligence (AI) models have gained unparalleled visibility in all sorts of domains ranging from business applications, drug discovery, financial trading, cyber security, manufacturing industry, IOT, simulator building, etc. [33, 34]. As compared to other ML models, an ANN model (also referred to as the feed forward neural network model) typically gives highly accurate prediction [35], but suffers due to overfitting and lacks in terms of the explainability.

The basic idea behind the formulation of an ANN model is to create a nested structure from the set of observable inputs to output via latent variables. In a typical ANN architecture, the input layer consists of all observable predictors which are combined and passed to latent variables by applying an activation function. We implemented the model in *Python* using **Keras** and **TensorFlow**. The tuning parameters (number of hidden layers and number of latent variables within a hidden layer) were optimally chosen using cross-validation within a simulation study. Rectified linear unit (ReLU) activation function was used for the hidden layers, whereas a linear activation function was used for the output layer.

#### 3.2.4 Support vector regression (SVR)

SVR, proposed by Vapnik et al. [36], attempts to find a hyperplane that can adequately capture the relationship between the response and the covariates. If the most optimal hyperplane is not a good enough regressor in the original input space, then a powerful kernel is used to transform the inputs to higher dimensional feature space. The model is fitted in this feature space to obtain more accurate predictions.

We used **scikit-learn** library in *Python* to fit the SVR model. Among the three popular kernels (linear, RBF and polynomial), RBF turned out to be the most optimal according to cross-validation with MSE criterion. The other hyper-parameters which were optimally found using a grid search, are (a) the regularization constant—*C*, which controls the trade-off between maximizing the margin and minimizing the training error; (b) shape parameter—*γ*, which determines the shape of the regression curve; and (d) the margin around the regression curve *ε*.

### 3.3 New hybrid model

We now propose an innovative two-stage hybrid model which properly accounts for the unusual spikes at 0 and 20 in the distribution of renewal life of patents (as shown in Fig 1). The first stage refers to accurate classification of the patents into three groups: never renewed (*Y*_*i*_ = 0), matured (*Y*_*i*_ = 20) and expired patents (0 < *Y*_*i*_ < 20). Table 3 presents the actual counts of the patents in the three categories for our data. We used a support vector machine-based classifier (SVC) for this task. Subsequently, a binomial regression model is used for further predicting the renewal life of expired patents.

**Table 3:** Distribution of patent counts from the cleaned data with respect to renewal life category and train-test splitting.

Patent category	Label	Train data	Test data	Total
Never renewed	1	4590	1147	5737
Expired	2	16526	4166	20692
Matured	3	2200	516	2716
Total		23316	5829	29145

To arrive at an optimal SVC model, cross-validation and grid search approach similar to Section 3.2.4 was followed. We considered (a) the three kernels: linear, polynomial and RBF; (b) regularization parameter *C* ∈ {0.1, 1, 10}; (c) the shape parameter *γ* ∈ {0.01, 0.1, 1}; (d) degree of the polynomial *d* ∈ {2, 3, 4}, and root mean square error (RMSE) for model ranking. For our Indian patent data, the optimal SVC model using 5-fold cross-validation corresponds to the linear kernel with regularization parameter value *C* = 0.1 and *γ* = 0.01.

The predicted value of renewal life for the patents classified as Label 1 and Label 3 are zero and twenty years, respectively. The patents which gave the predicted class label as 2 were modelled further using the binomial regression model discussed in Section 3.1. The predicted renewal life for these patents were obtained as per the binomial regression model. Subsequently, the predicted values of patent renewal life were utilized for calculating the goodness of fit measures (RMSE and Pearson’s coefficient).

## 4 Results and discussion

All models presented in Section 3 are now discussed and compared at length. For a fair comparison all the model fits assume the same (80-20) train-test splits. In the first stage of the hybrid model, we are solving a classification problem, whereas in the second stage, it is a regression problem. At the end, we are predicting renewal years in the range {0, …, 20}. Moreover, each competitor presented in Section 3 solves the renewal life prediction problem as a regression exercise. Therefore, the performance comparison is quantified with respect to root mean square prediction error (RMSPE) and Pearson correlation given by:

$$RMSPE=[\frac{1}{n}\sum_{i=1}^{n}{\left(y_{i}-\hat{y}\left(x_{i}\right)\right)}^{2}{]}^{1/2}\;\text{and}\;Cor\left(Y,\hat{Y}\right)=\frac{Cov(Y,\hat{Y})}{sd\left(Y\right)\;sd\left(\hat{Y}\right)}.$$

Note that the RMSPE is supposed to be minimized and the Pearson correlation has to be maximized.

**Fig 7:**
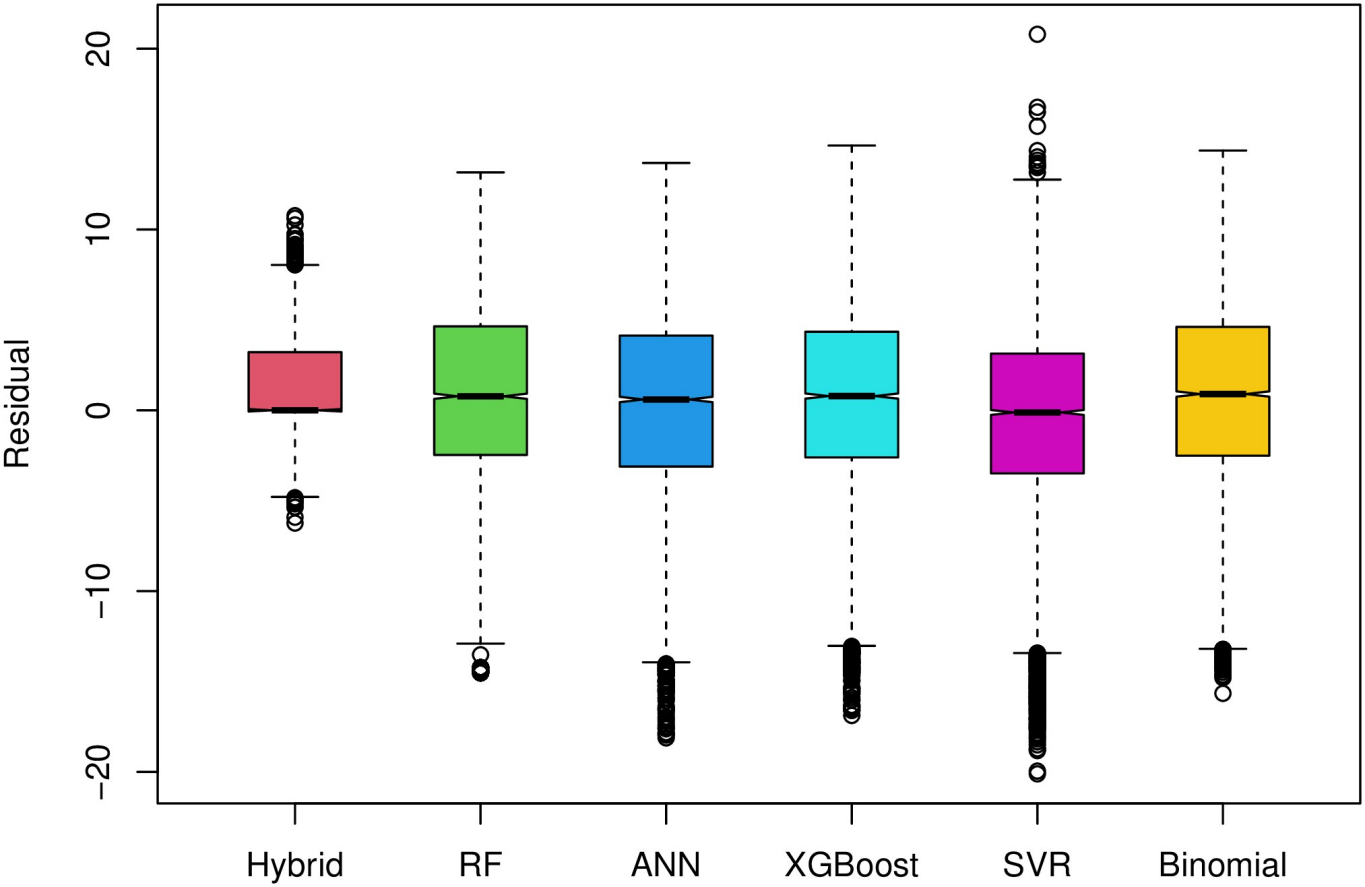
Comparative boxplot of the prediction residuals for the test data sets obtained from the six predictive models.

The distribution of prediction residuals obtained from different models for the test data are depicted in Fig 7.

Fig 7 shows that the range of the boxplot for the hybrid model is much narrower as compared to other competitors. Table 4 values indicate that the hybrid model demonstrates 90% accuracy as compared to the best alternative (XGBoost) with only 40% accuracy quantified by the Pearson correlation. The RMSPE values for the hybrid model is also almost half as compared to the competitors. That is, the proposed two-stage hybrid model outperforms the state-of-the-art ML models by a significant margin when measured as per the RMSPE and Pearson correlation between the actual and the predicted renewal life of the test data.

**Table 4:** Prediction accuracy comparison of all model fits with respect to (80-20) train-test split.

Models	RMSPE		Pearson correlation	
	Train	Test	Train	Test
New hybrid	3.056	3.043	0.904	0.902
Random Forest	6.066	6.048	0.314	0.314
ANN	5.917	5.965	0.378	0.354
XGBoost	5.855	5.903	0.404	0.376
SVR	6.106	6.168	0.348	0.322
Binomial	6.107	6.072	0.294	0.302

The binomial regression part of the hybrid model can be used to assess the significance of the predictors. Table 5 summarizes the significance of patent features in determining the renewal life of expired patents. Clearly all features are significant with the reference level of significance *α* = 0.01, but in the order of preference, the most significant would be “Filing year” and the least significant is the “Number of Claims”.

**Table 5:** Estimates and significance of the predictors obtained from the binomial regression part of the hybrid model.

Independent Variable	${\hat{\beta }}_{j}$	SE(${\hat{\beta }}_{j}$)	Z-stats	P-value
Intercept	0.1842	0.0208	8.855	≈ 0
FilingYear	-1.0333	0.0143	-72.2587	≈ 0
NumOfClaims	-0.0383	0.0147	-2.605	0.0045
InventorSize	0.1728	0.0117	14.7692	≈ 0
FamilySize	0.4214	0.0189	22.2963	≈ 0
TechScope	0.1955	0.0149	13.1208	≈ 0
GrantLag	0.2578	0.0200	12.8900	≈ 0
Ownership	0.1719	0.0098	17.5408	≈ 0
Patentee Indiv.	-0.2038	0.0110	-18.5272	≈ 0
Patentee Insti.	0.4257	0.0125	34.0560	≈ 0
Techclass Elect.	0.0683	0.0084	8.1309	≈ 0
Techclass Instr.	-0.0462	0.0111	-4.1621	1.59e-05
Techclass Mech.	-0.0249	0.0080	-3.1125	0.0009
Techclass Other.	-0.1518	0.0152	-9.9868	≈ 0

## 5 Conclusion

The main purpose of this paper was to find an accurate model for predicting the renewal life of Indian patents. We implemented several state-of-the-art ML models and a suitable statistical regression technique called the binomial regression for building the prediction model. However, the prediction accuracy values were very low. Although the renewal life ranges between 0 and 20, the presence of spikes at the two extremes poses a great challenge for modelling techniques.

In an attempt to fill this research gap, we proposed an innovative two-stage hybrid model. The first stage classifies the patents in three categories, “never renewed”, “expired” and “matured”, with the predicted class label for never renewed and matured being 0 and 20 respectively. Next, all expired patents are processed to fit a binomial regression model for predicting their renewal life. When testing the relevance of the patent value indicators, we found that patent claims are the least significant (consistent with the findings of Hu et al. [37]), but interestingly the results also reveal that newer patents tend to have shorter renewal life. The proposed hybrid model demonstrates 90% accuracy as compared to the best alternative with only 40% accuracy.

A future study can use the similar model to apply on a varied collection of patent value predictive indicators such as collaboration between industry and academia, collaboration across the boarder, technology (complex vs discrete), and available patents on the similar line (technology similarity). This model can also be used to predict the possibility of patent commercialization in the future across the technology. Information on the essence of technology, the cost dimension (transfer cost, reference cost, and research and development cost), the product market, and the technology market (number of suppliers, number of demands, commercial level), for example, could also be useful in predicting more accurate renewal life in the very early stage of the patent. Another future direction is to include recent patents as well, as the most recent patents may still be active and hence one may have to include survival analysis-based models with right-censored renewal life data.

*Practical implications*: Robust patent systems protect innovations by granting exclusive intellectual property rights to new ideas and initially eliminating trivial patents. This solves the problem of inducing the optimum rate of technological change. We assume that the patent system is an effective tool for promoting technological change; the question is how to make it more efficient. In the pursuit of these questions, predictive analysis of patent life offers a solid solution to very practical issues. Begin by enhancing the patent system to remove low-quality or frivolous patents during application. Secondly, it allows businesses, especially startups, to forecast the duration of a patent using the deterministic estimation method outlined by [11, 12]. The government can assess technological advancement by analysing patent longevity in addition to patent counts. This allows them to develop policies based on the pace of technological development in different sectors. The model effectively addresses the three problems. Initially, eliminate low-quality patents from the system. Secondly, it assists businesses in analyzing their patent portfolio and allows them to negotiate with companies interested in their portfolio. Thirdly, it assists the government in formulating policies based on the predictive outcomes of the patent life cycle. Moreover, it is a beneficial approach to decrease the deadweight loss caused by frivolous patenting and enhance the efficiency of the patent system to some degree.
